# Deformation Behaviour and Damage Evolution of Carbonaceous Phyllite under Cyclic Triaxial Loading

**DOI:** 10.3390/ma16134612

**Published:** 2023-06-26

**Authors:** Helin Fu, Kaixun Hu, Yue Shi, Jie Li, Yimin Wu

**Affiliations:** 1 School of Civil Engineering, Central South University, Changsha 410075, China; 2 National Engineering Laboratory for High Speed Railway Construction, Central South University, Changsha 410075, China

**Keywords:** carbonaceous phyllite, cyclic triaxial loading, deformation behaviour, damage analysis, failure mode

## Abstract

Rocks present complex deformation behaviours and damage processes under triaxial cyclic loading—a subject not yet sufficiently researched. This paper performed triaxial multistage constant-amplitude cyclic loading experiments under different confining stresses on carbonaceous phyllite. The degradation process is analysed by investigating the variation of elastic modulus *E_S_*, Poisson’s ratio *υ*, irreversible strain *ε^irr^* and energy. Moreover, the rock’s failure mode is explored from both macro and micro perspectives. The results showed that the increase in stress level caused the decrease of *E_S_* in a step-like form, and the constant-amplitude cyclic loading in each stress level caused a slow decrease of *E_S_*, while the *υ* increased with stress level and constant-amplitude cycles in a similar form. *ε^irr^* accumulated rapidly at first and then slowly at each stress level; the stress level and irreversible axial strain are related by an exponential function. In terms of energy evolution analysis, the damage to rock can be represented by the cumulative damage energy, there were deceleration accumulations and stability accumulation stages of damage at all stress levels, and an acceleration accumulation stage occurred when the rock was close to failure. The failures of rock under cyclic loading are mainly shear failures, accompanied by grain crushing.

## 1. Introduction

Due to the global economy’s continuous growth and the need to speed up the urbanization process, many engineering facilities related to the geotechnical field are being planned and constructed worldwide [1,2], such as reservoirs, railway tunnels and gas storage caverns, which are often faced with cyclic loading. Cyclic loading often causes the accumulation of internal damage and a reduction in strength with regard to rock [3,4,5]. Therefore, studying how cyclic loading affects the deformation and damage of rock is significant to maintain the safety and stability of rock engineering projects.

Extensive research has been conducted in the past few decades on the response characteristics of rock under cyclic loading. These studies are mainly performed with respect to the following three types of cyclic experiments: constant-amplitude cyclic loading experiments, multistage constant-amplitude cyclic loading experiments, and increasing-amplitude cyclic loading experiments [6,7,8]. These experiments consider the effects of frequency [9,10], amplitude [11,12,13], average stress [14], waveform [15,16,17] and other factors. For the constant-amplitude cyclic loading experiment, the rock’s compressive strength under repeated loading is typically lower than its monotonic compressive strength. [18]. Irreversible strain accumulates in each cycle, and the accumulation rate changes from slow to fast with the development of the cycle. There is little effect of the cycle on the degradation of elastic modulus [19,20,21,22], thus only a limited decrease is detected in some studies [23,24]. The higher the frequency of cyclic loading, the more cycles required for rock failure, and the greater the corresponding limit axial strain [3]. For the multistage constant-amplitude cyclic loading experiment, Jia et al. [25] conducted the corresponding experiments on fine-grained sandstone and concluded that the elastic modulus has different variation laws with cycles in regard to different stress level, so does Poisson’s ratio. Heap and Faulkner [26] demonstrated that increasing stress levels can cause a reduction in sample stiffness, as evidenced by a decrease in elastic modulus and an increase in Poisson’s ratio. Increasing-amplitude cyclic loading experiments are often used for damage-controlled analysis. These experiments found that the irreversible strain and Poisson’s ratio increase slowly with the cyclic loading [27,28], while Young’s modulus exhibits the opposite trend [29,30], which is related to rock expansion and crack propagation behaviour.

In addition to the change in deformation behaviours, fatigue damage accumulation is also significant during cyclic loading. The fatigue indicators often considered the inclusion of residual axial deformation [23,31], residual volumetric deformation [15], elastic/secant modulus [32,33] and energy dissipation [34]. The reduction in the elastic modulus and the accumulation of residual deformation are external factors contributing to fatigue damage, while energy dissipation is an internal factor. This enabled many scholars to conduct damage analysis in terms of energy. Many [35,36] adopted normalized dissipated energy to represent the damage of rock and observed that the resulting damage curve can be divided into three stages, which were deceleration accumulation, stability accumulation, and acceleration accumulation, indicating a certain damage evolution law.

However, the tests mentioned above still need to be further improved. Regarding the selection of loading tests, studies on rock deformation behaviour have primarily focused on uniaxial monotonic and cyclic loading, while the rocks involved in applied engineering are typically in three-dimensional stress states. Additionally, the cyclic triaxial loading of rocks is mainly concentrated at low confining pressures *σ*_3_ [25]. In fact, many engineering projects are constructed with high burial depths, causing the rock to face high confining pressure, such as in the case of some high geo-stress tunnels on the Sichuan–Tibet Railway in China. Under such high confining pressure, the evolution of deformation will be different from that of damage, and the cyclic loading state of rock is complex. For the study of energy, it is rare to distinguish the damping energy consumed by cracks of rock due to the viscoelasticity, resulting in a certain deviation in the law of energy evolution.

This paper aims to investigate the deformation characteristics and damage evolution of carbonaceous phyllite under triaxial cyclic loading. First, the compressive strength measured by the monotonic triaxial tests was used to design the stress levels of cyclic loading. Second, triaxial multistage constant-amplitude cyclic loading tests were conducted under various confining stresses, and the mechanical parameters, irreversible strain, volumetric strain and energy evolution were analysed. Finally, the macro failure morphology of the specimen was recorded, and its micromorphology was tested by scanning electron microscopy (SEM), thus the corresponding failure mechanism was investigated. This study intends to offer insights into the design, construction, and maintenance of rock projects such as tunnel construction.

## 2. Experimental Method

### 2.1. Experimental Specimens and Facilities

**Figure 1:**
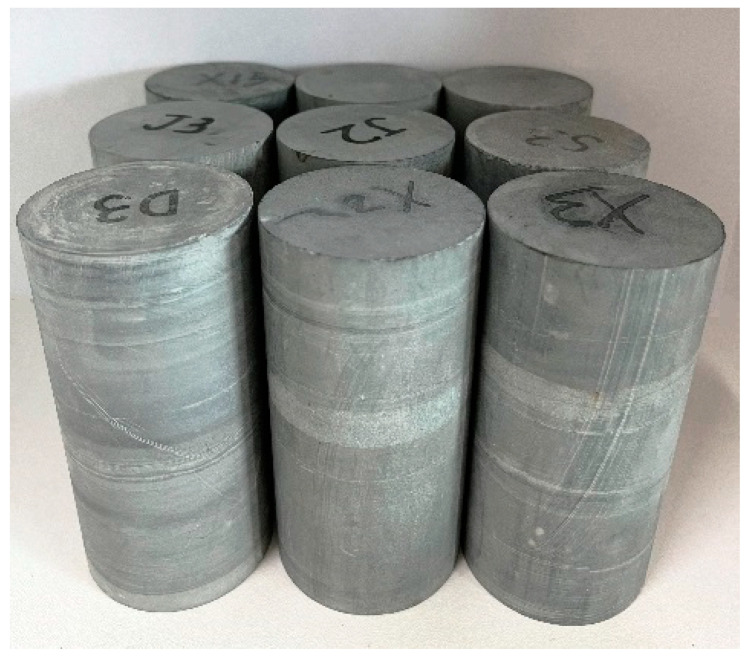
Display of carbonaceous phyllite specimens.

The carbonaceous phyllite specimens used in the experiment were taken from the surrounding rock of the Hufengling Tunnel of the Harbin–Mudanjiang high-speed railway in Heilongjiang Province, China. The Hufengling Tunnel, with a length of 8755 m, is the longest high-speed railway tunnel located in the cold regions of China. Carbonaceous phyllite is a kind of gray-black metamorphic rock with phyllitic structure. Its metamorphic degree is between slate and gneiss, the lithology is soft, and it is prone to argillation and softens when encountering water. Because of the poor mechanical properties, the tunnel with carbonaceous phyllite surrounding rock under cyclic dynamic loading is subjected to some diseases, such as the bulge of the inverted arch, lining cracking and even collapse, which are difficult to repair. Therefore, the carbonaceous phyllite was chosen to test the mechanical property; the dry blocks were cut and polished to prepare cylindrical samples, with each sample prepared according to the International Society for Rock Mechanics (ISRM) test guidelines, with a diameter of 50 mm and a length of 100 mm. However, there were some errors in the actual operation process. The average diameter and average length of the final batch of test specimens were 49.9 mm, 100.3 mm, respectively. The phyllite was characterized by bedding, and the bedding plane of the specimen was perpendicular to the axial direction. The test specimen is displayed in Figure 1, and related physical and mechanical parameters are tested first, as shown in Table 1, in which *ρ* is the density, *E_S_* is the elastic modulus, *υ* is Poisson’s ratio, *c* is the cohesion, *φ* is the internal friction angle and *σ_c_* is the uniaxial compressive strength.

**Figure 2:**
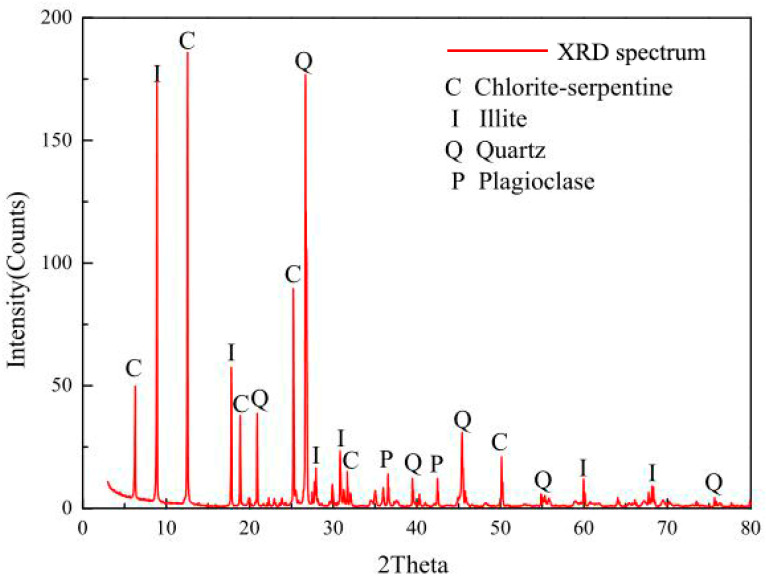
Diffraction pattern of mineral composition.

**Figure 3:**
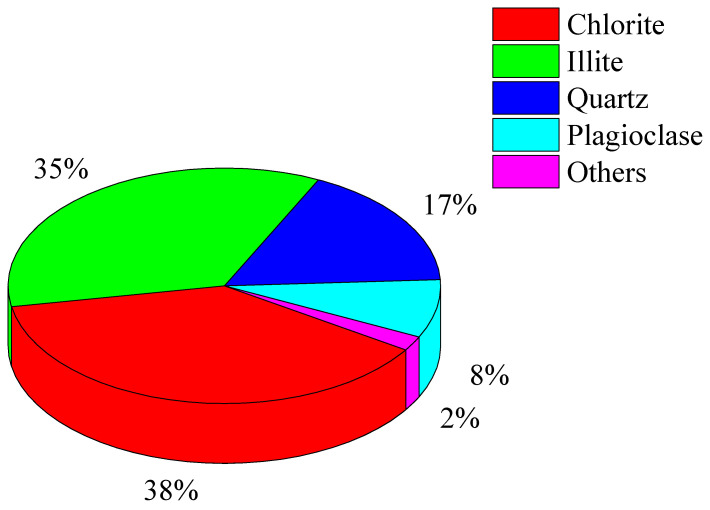
Mineral composition of phyllite.

The composition of the specimens was explored through mineral composition analysis (XDR), as shown in Figure 2 and Figure 3. The findings indicate that the specimens contained high amounts of hydrophilic minerals, which are susceptible to water; in other words, it is easy to soften when encountering water. As a result, the frozen environment in the cold region will strengthen the carbonaceous phyllite, while the thawed environment will soften the rock. This means that they may be influenced by the freeze–thaw cycle in the cold region.

**Figure 4:**
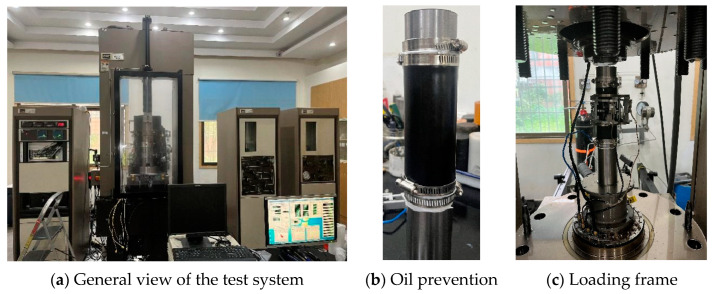
The MTS-815 Test System.

The loading facility adopted the MTS815 test system (Meters Industrial Systems, Inc., Cleveland, NC, USA), as shown in Figure 4a. The experimental setup consists of a static loading frame, dynamic loading frame, and data acquisition system, which adequately fulfilled the requirements for conducting both monotonic and cyclic loading tests on rock. To prevent errors in the results caused by oil seepage, oil leakage prevention measures were adopted (Figure 4b). The loading frame of the equipment is shown in Figure 4c; the maximum value of compression load, tensile load and confining pressure can reach 4600 kN, 250 kN and 140 MPa, respectively. In addition, the confining pressure was achieved through applying static oil pressure by confining pressure intensifier, which is precisely controlled by the digital system.

The axial dynamic loading frame was hydraulically driven, and the loading rate could be controlled by controlling the load, stress, and displacement. The sampling rate of the data acquisition frame can reach a maximum of 6 kHz, allowing for the recording of stress and strain information, in which the latter was measured by extensometers in the axial direction and lateral direction.

### 2.2. Experimental Schemes

In terms of the railway tunnel, the surrounding rock at the tunnel base often faces dynamic train loads, which contain constant-amplitude cyclic loads with different stress levels. The cyclic load is a kind of repeated load, which is determined by the number of repeat loading, frequency, cyclic stress variation (including square, triangle or sinusoidal) and stress level (including the mean stress and stress amplitude). This kind of cyclic load will induce the accumulated deformation or damage of surrounding rock, and the damage evolution and fatigue limit are mainly influenced by the stress level.

To study the property attenuation of carbonaceous phyllite under cyclic loading, and explore the influence of different stress levels, multistage constant-amplitude cyclic loading tests were conducted. In addition, the stress levels are determined by referring to compressive strength, thus the monotonic compression tests were carried out first. The confining pressure for the monotonic and cyclic loading tests was applied at three representative values (10 MPa, 30 MPa, and 50 MPa), which represent low, medium and high confining pressures. Each test was repeated three times to eliminate the influence of the discreteness of the specimens, thus there are nine rock samples for monotonic loading tests and another nine rock samples for cyclic loading tests. The loading schemes were as follows.

In the monotonic loading test, the confining pressure was initially provided at a rate of 0.2 MPa/s, followed by a stabilization period. Then, the deviatoric stress was provided in the axial direction at a rate of 0.02 mm/min until the rock failure occurred.

**Figure 5:**
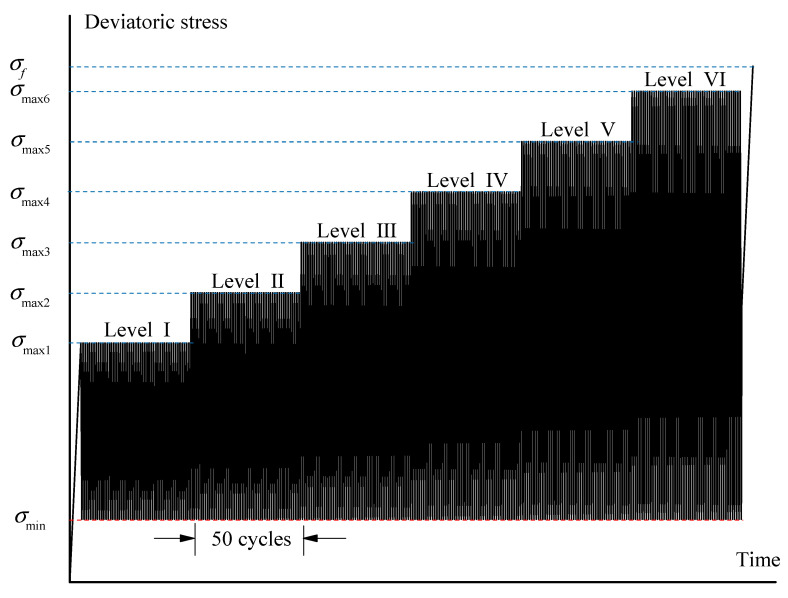
Cyclic loading path (*σ_f_* is the compressive stress of monotonic loading under the same confining pressure).

In the cyclic loading test, the axial cyclic load waveform was a triangle (Figure 5), and the specific test process was as follows.

(1) The confining pressure was provided at a constant rate of 0.2 MPa/s.
(2) Applying the deviatoric stress in the axial direction at a rate of 0.02 mm/min until the first level. Then, 50 constant-amplitude cycles were performed within the level with a frequency of 0.1 Hz.
(3) The subsequent multistage constant-amplitude cyclic process was applied in the same manner as step two.
(4) After the cyclic loading process, the sample was loaded monotonically at a rate of 0.02 mm/min until the rock failure occurred.

## 3. Experimental Results

### 3.1. Stress–Strain Curve

**Figure 6:**
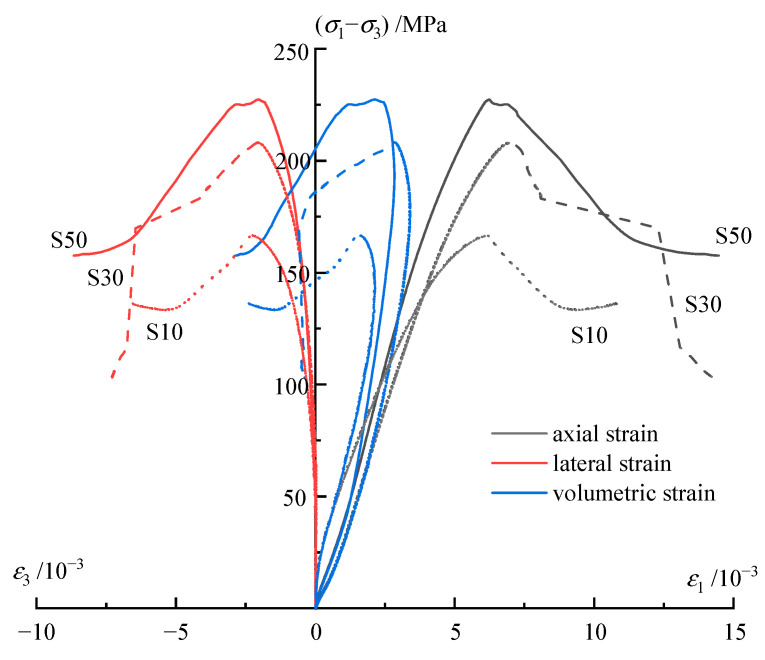
Stress−strain curves of phyllite under monotonic loading.

Monotonic compression tests were carried out on carbonaceous phyllite samples to provide a reference for designing the stress level of multistage constant-amplitude cyclic loading experiments. In Figure 6, the stress–strain curves with various confining stresses under monotonic loading are shown, in which *ε*_1_, *ε*_3_ are the axial strain and lateral strain respectively, *σ*_1_, *σ*_3_ are the axial stress and lateral stress, respectively. The mechanical parameter results are compiled in Table 2, in which *σ_f_* is the compressive stress under the monotonic loading, *ε_f_* is the corresponding strain. According to the test results and the achievements of Liu et al. [37], the damage process underwent the following five stages. First, in the compaction stage, the initial compression process closes the cracks. Second, in the elastic deformation stage, the experimental curve exhibits a linear trend. Third, in the crack generation and extension stage, microcracks begin to develop with increasing stress, whose number, formation rate, and density gradually increase. Fourth, in the failure stage, the specimen fails once the deviatoric stress exceeds the compressive strength. Fifth, in the postpeak stage, the microcracks of the rock gradually become connected, which causes the rock to exhibit softening characteristics; as a result, the rock strength remains basically unchanged, but the deformation continues to increase.

The mean stresses and stress amplitudes for each stress level in the cyclic loading test were designed based on the compressive strength obtained from the monotonic loading test. Considering that the cyclic load approximating *σ_f_* always causes greater damage compared to early cyclic loading, the third stage (crack generation and extension stage) and the fourth stage (failure stage) attract more attention in this paper. Therefore, the multistage constant-amplitude cyclic loading was mainly arranged in the abovementioned two stages. Before the cyclic loading, the rock sample was monotonically loaded to the third stage. After the cyclic loading, the sample was expected to approach failure, thus the monotonic loading was designed again until the sample failed. Taking the efficiency of the experiments into consideration, six groups of cycles with different stress levels were allocated during the two stages, which can provide sufficient data for researching the effect of cyclic loading and stress levels, the mean stresses and stress amplitudes for each stress level are presented in Table 3, in which *σ_min_* is the minimum axial deviation stress of each cycle, and *σ_max_* is the maximum axial deviation stress of the cycle.

**Figure 7:**
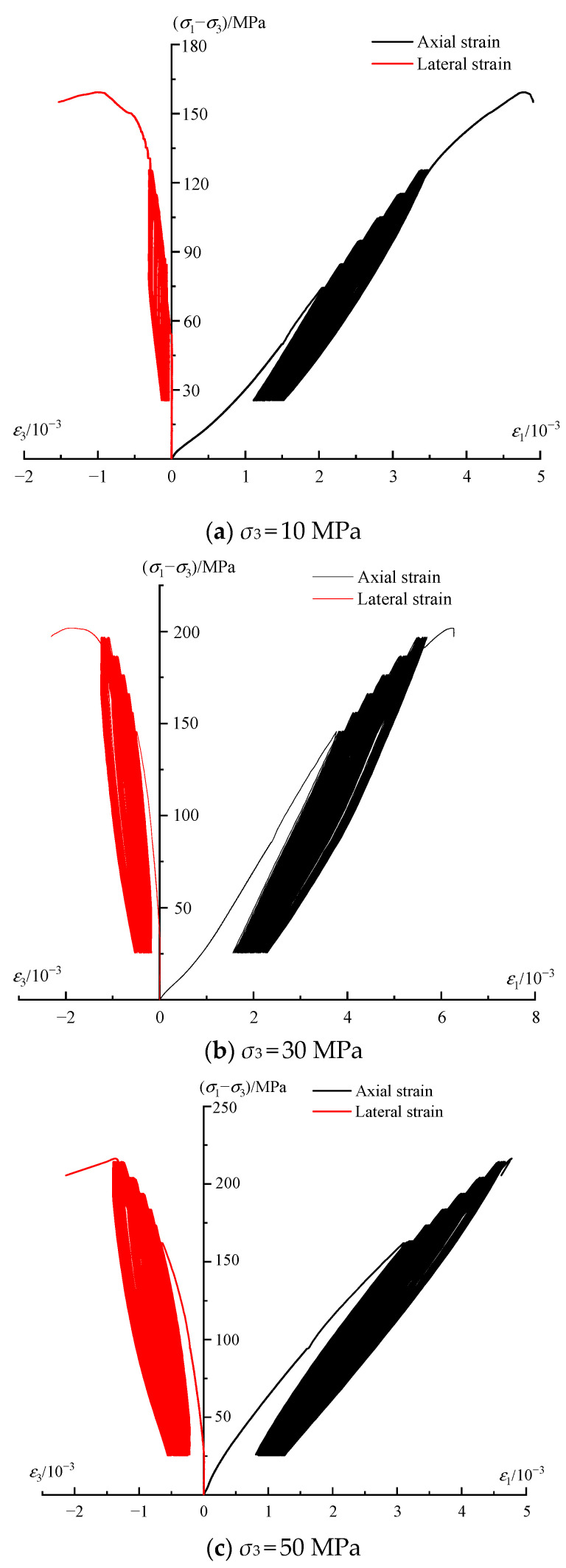
Stress−strain curves of phyllite under cyclic loading.

The stress–strain curves for specimens under three different confining stresses during the cyclic test are illustrated in Figure 7, where the three curves demonstrate a similar trend. The hysteretic loops in each stress level, apart from the last stress level, follow a pattern of opening–closing, and the pattern of the last stress level is changed to opening–closing–opening. The result can be explained through the energy perspective by referring to the work of Cerfontaine and Collin [38]. At the beginning of each stress level, a considerable part of the energy is used to develop inner cracks, which corresponds to the sparse state of hysteretic loops; after the first several cycles, there is little energy to be consumed because the cracks are closed and basically remain stable, which corresponds to the dense state of hysteretic loops. With regard to the stress level that approaches the compressive strength, multiple cycles will enable rock to reach the strain limitation; thus, the closed cracks will continuously extend and propagate, corresponding to the second sparse state of the hysteretic loops at the final stress level. In addition, it is also observed that the hysteretic loops within the *i* + 1th stress level are sparser than those of the *i*th stress level. As the stress level increases, the rate of plastic strain also increases proportionally.

### 3.2. Variation of Mechanical Parameters

The elastic modulus *E_S_* and Poisson’s ratio *υ* are two important mechanical parameters that are affected by the development, extension, and coalescence of cracks, so they are capable of reflecting the deformation behaviours to some degree. The calculations of *E_S_* and *υ* are expressed as Equations (1) and (2), respectively, as follows:

(1) $$E_{S}=\frac{\Delta \left(\sigma_{1}-\sigma_{3}\right)}{\Delta \varepsilon_{1}-\Delta \varepsilon_{1}^{irr}}$$

(2) $$\upsilon =\frac{\Delta \varepsilon_{3}-\Delta \varepsilon_{3}^{irr}}{\Delta \varepsilon_{1}-\Delta \varepsilon_{1}^{irr}}$$

where Δ(*σ*_1_ − *σ*_3_) is the value between the maximum value and minimum value of deviation stress in each cycle, Δ*ε*_1_ and Δ*ε*_3_ are the change values of axial strain and lateral strain in each cycle, respectively, and Δ*ε*_1_*^irr^* and Δ*ε*_3_*^irr^* are the increasements of irreversible axial strain and irreversible lateral strain in each cycle, respectively.

**Figure 8:**
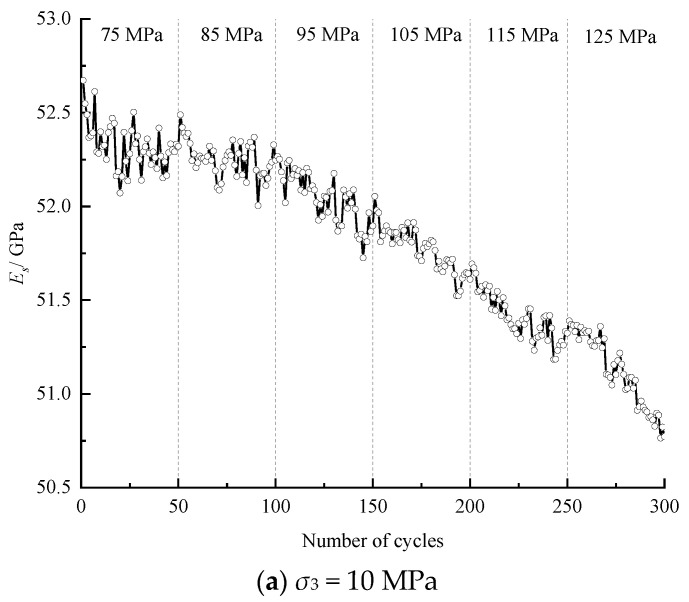

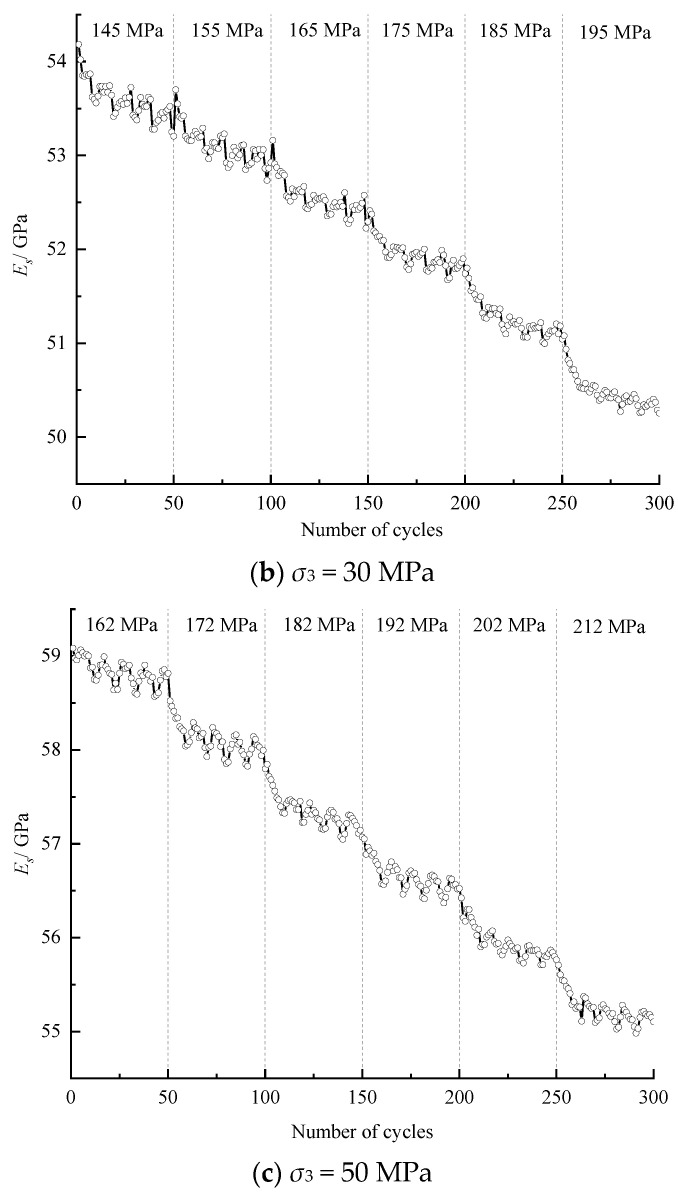
Evolution of the elastic modulus with an increasing number of cycles.

The *E_S_* evolutions of the specimens under different confining stresses during the entire cyclic process are shown in Figure 8, which indicates that the stress level and multiple cycles in each level both affect the variation in *E_S_*. On the one hand, every increasing stress level will prompt *E_S_* to fall to a certain extent, and this effect often occurs in the first several cycles of each stress level. On the other hand, after the fall, the subsequent multiple cycles within each stress level will induce an approximately linear decrease in *E_S_*, and the rate of decrease increases with the increase in the stress level. The evolution process of *E_S_* reveals that the increasing stress level will cause relatively greater damage to the specimen, while the equal amplitude cyclic loading and unloading induces a gradual attenuation of the specimen.

**Figure 9:**
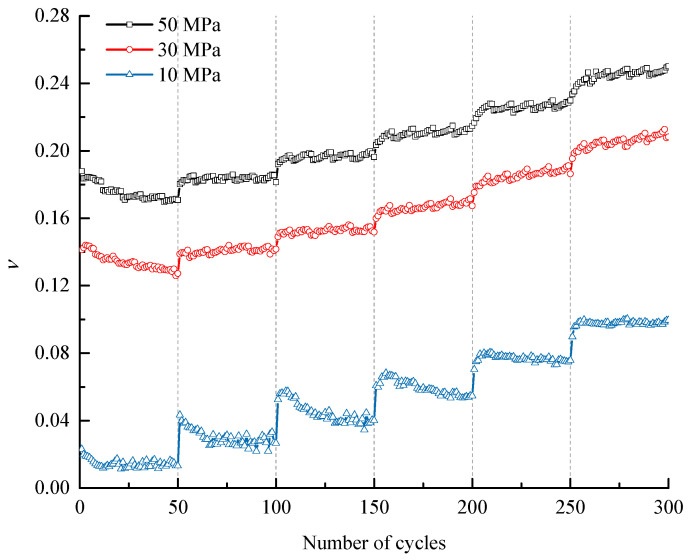
Variation of Poisson’s ratio with the number of cycles under different confining pressures.

**Figure 10:**
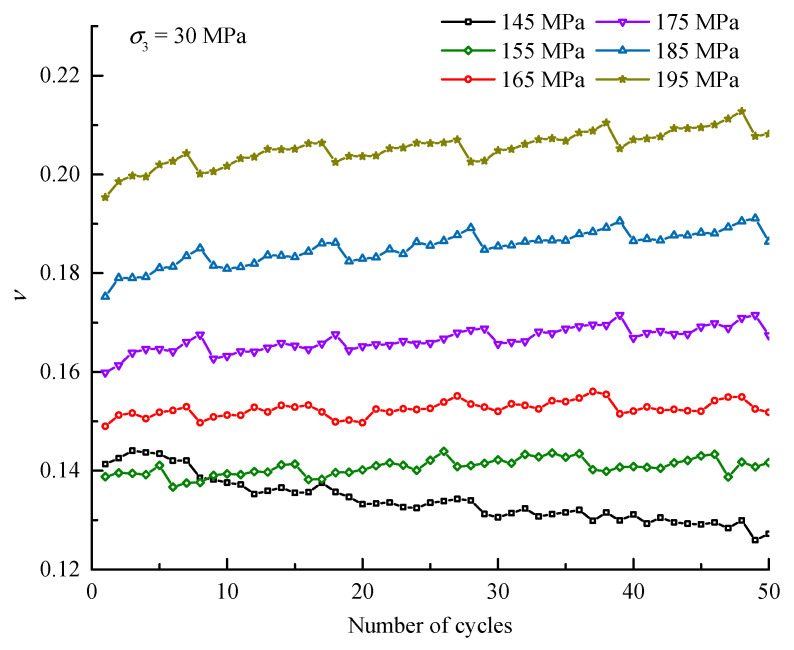
Variation of Poisson’s ratio during each stress level (*σ*_3_ = 30 MPa).

The *υ* evolutions of the specimens under different confining stresses during the entire cyclic process are shown in Figure 9, and the detailed variations of *υ* with the number of cycles within each stress level are extracted and displayed in Figure 10. The findings show that *υ* has a similar evolution law to *E_S_*. On the one hand, every increasing stress level will prompt *υ* to increase to a certain extent, which occurs at the first cycle of each stress level. On the other hand, equal amplitude cyclic loading and unloading within most stress levels will induce a slow increase in *υ*, and the increase rate is positively related to the stress level. In addition, a downwards trend of *υ* is obviously observed in the first three stress levels under *σ*_3_ of 10 MPa and in the first stress level under *σ*_3_ of 30 MPa and 50 MPa, which may be attributed to the fact that the rock particles are relatively loose when the stress level is low, in which the initial cycles enable the particles to gradually become dense; thus, the lateral deformation ability decreases.

### 3.3. Variation of Irreversible Axial Strain and Lateral Strain

**Figure 11:**
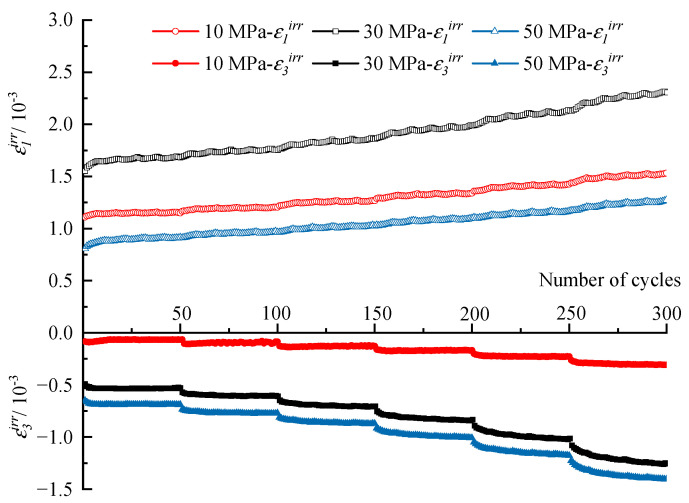
Variation of irreversible strain with the number of cycles.

**Figure 12:**
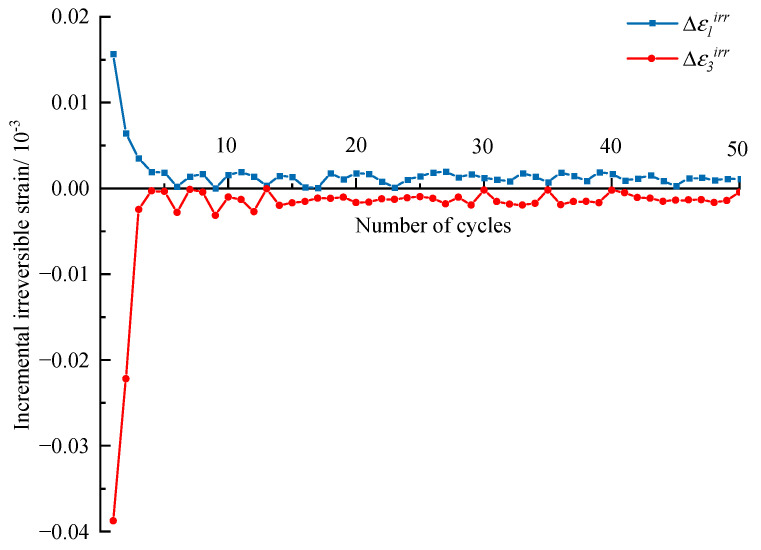
Evolution of incremental irreversible strains with cycle numbers at the second stress level. (*σ_3_* = 10 MPa).

First, the evolution of irreversible strain during the entire cyclic process under different confining stresses is discussed here. The results are shown in Figure 11, in which *ε*_1_*^irr^* is the irreversible axial strain and *ε*_3_*^irr^* is the irreversible lateral strain. *ε*_1_*^irr^* and *ε*_3_*^irr^* increase step by step with increasing stress levels, and the variation laws within different stress levels are similar. To explore the variation law more clearly, the increments of *ε*_1_*^irr^* and *ε*_3_*^irr^* in terms of each cycle within one stress level are extracted and shown in Figure 12. The first several cycles contribute most of the irreversible strain to the stress level, and then the increments of *ε*_1_*^irr^* and *ε*_3_*^irr^* become small and stable for the subsequent cycles of the stress level. Overall, the irreversible strain within one stress level consists of two parts, where one is the proliferation part in the first several cycles, the other is the slowly increasing part in subsequent cycles, and the increase rate is larger with the increasing stress level.

**Figure 13:**
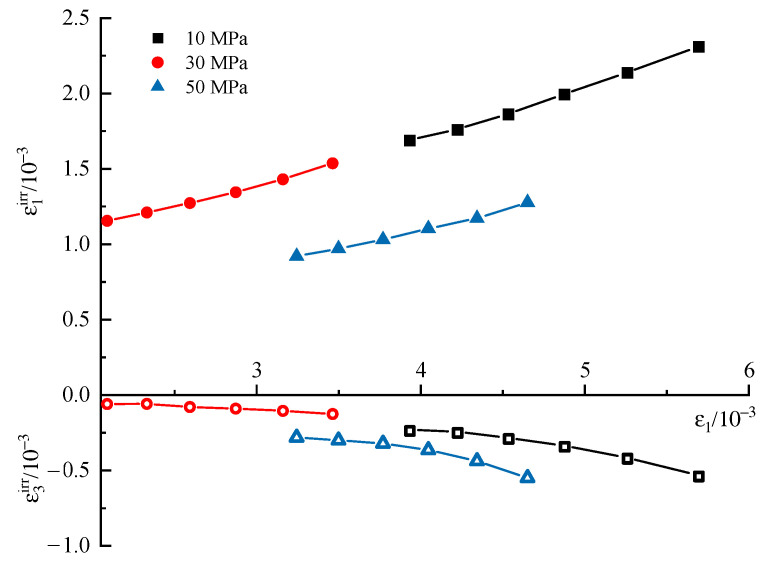
Development of irreversible strain with the axial total strain.

Figure 13 displays the relationship between *ε*_1_*^irr^*, *ε*_3_*^irr^*, and *ε*_1_. *ε*_1_*^irr^* increases linearly with *ε*_1_, while *ε*_3_*^irr^* exhibits a trend of accelerating increase, which reveals that the plastic part of lateral strain increases faster than the elastic part.

**Figure 14:**
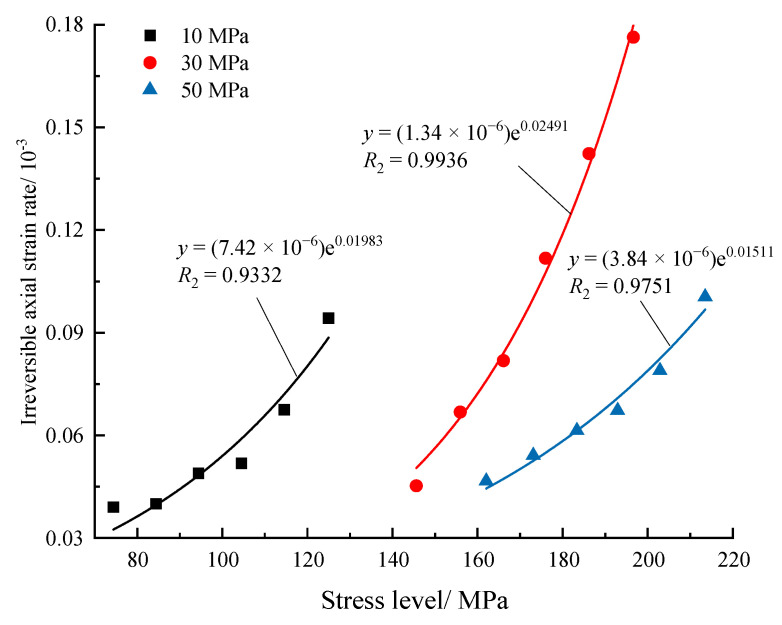
Development of increments of irreversible axial strain with stress level.

Figure 14 displays the accumulated *ε*_1_*^irr^* in 50 constant-amplitude cycles of different stress levels and shows that the higher the stress level is, the more *ε*_1_*^irr^* is generated in the same number of cycles, which reveals that the specimen is prone to fatigue damage under the high-stress level. In addition, according to the experimental results, the relationship between accumulated *ε*_1_*^irr^* and stress level can be well fitted by the exponential function, which is expressed as Equation (3).

(3) $$\Delta \varepsilon_{1}^{irr}=\alpha \cdot \exp(\beta \cdot \sigma )$$

It can be concluded that the number of cycles and the stress level significantly influence the irreversible strain. The evolution law of irreversible strain is similar to the typical creep curves of rock, which contain three stages called the deceleration accumulation stage, stability accumulation stage, and acceleration accumulation stage.

### 3.4. Variation of Volumetric Strain

**Figure 15:**
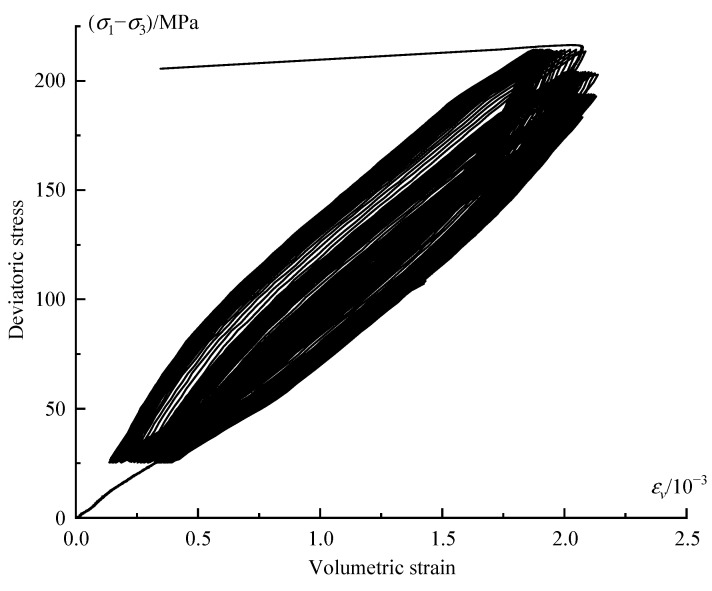
Variation curve of deviatoric stress–volumetric strain.

Figure 15 plots the deviatoric stress versus volumetric strain. In terms of the initial several stress levels, the specimen is mainly subjected to compaction, and the hysteretic loops are small in width and are close to each other. However, in terms of the higher stress level, the specimen exhibits dilation phenomenon, and the hysteretic loops become sparser.

**Figure 16:**
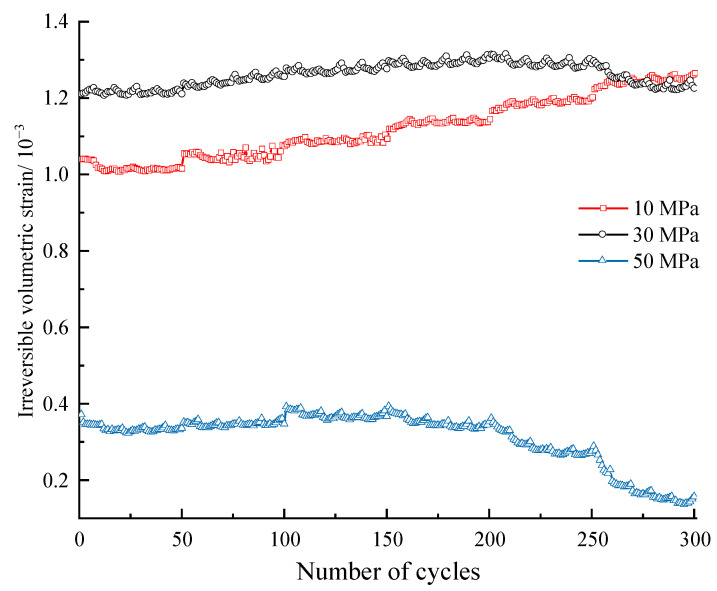
Variation of irreversible volume strain during the whole cyclic loading process.

**Figure 17:**
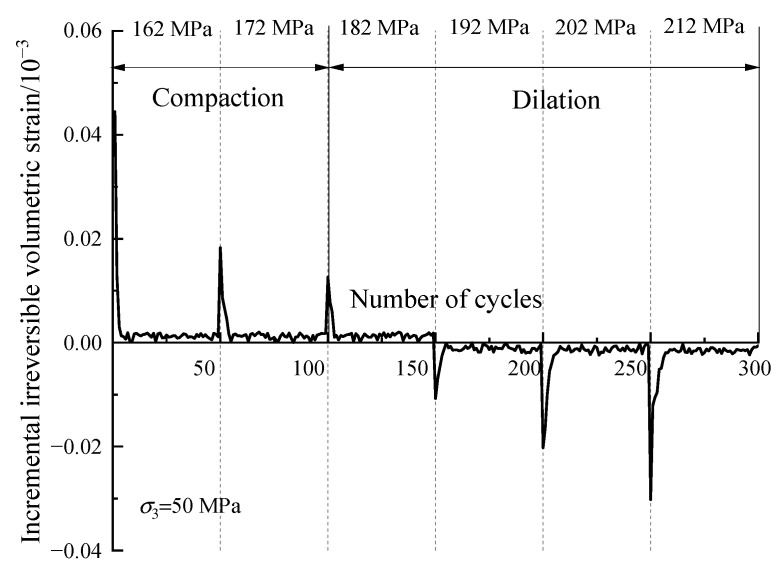
Variation of increment of the irreversible volumetric strain during the whole cyclic loading process.

Figure 16 plots the development of irreversible volumetric strain $\varepsilon_{v}^{iir}$ during the whole cyclic loading process. D10 specimen exhibits a trend of monotonic increment in terms of the irreversible volumetric strain, while that of the D30 and D50 specimens increase initially and then decrease. This is because the stress level designed is much lower than the compression strength in terms of D10 specimen, which does not enter the dilation stage. Taking the D50 specimen as an example, the evolution of the increment of $\varepsilon_{v}^{iir}$ is shown in Figure 17. The increment is positive during the first three stress levels, and the first few cycles in each stress level account for most of $\varepsilon_{v}^{iir}$, which means the specimen is in a compaction stage. As for the last three stress levels, the increment of $\varepsilon_{v}^{iir}$ is negative, which means the specimen is in a dilation state.

### 3.5. Assignment and Evolution of Energy under Cyclic Loading

#### 3.5.1. Assignment of Energy Considering the Damping Feature

**Figure 18:**
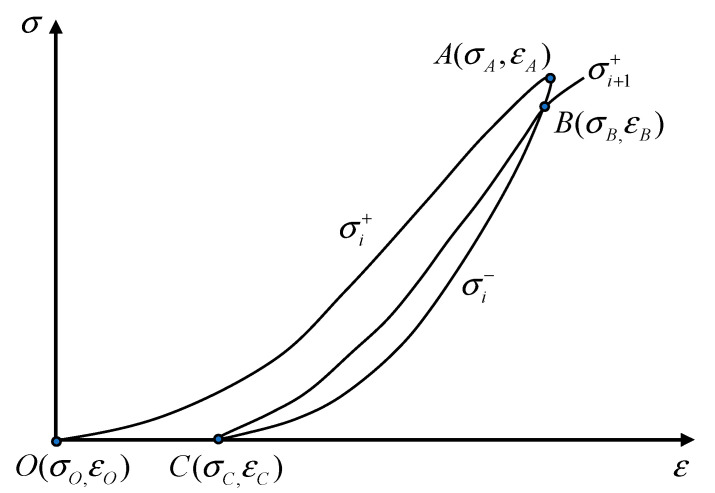
Schematic diagram of the energy calculation under cyclic loading and unloading.

The deformation of the rock specimen is accompanied by energy variation, which can reflect the damage to some degree. According to the first law of thermodynamics [39], the total input energy from the facility is transformed into elastic energy and dissipation energy of the specimen on the premise of eliminating heat loss, in which the elastic energy is stored in the form of elastic deformation, while the dissipation energy is consumed due to the plastic deformation of rock. The calculation of each energy type can be observed in Figure 18 and deduced by Equation (4).

(4) $$\left\{ \begin{array}{l} U_{i}=U_{ei}+U_{hi}=\int_{\varepsilon_{O}}^{\varepsilon_{A}}\sigma_{i}^{+}d\varepsilon_{i}\\ U_{ei}=\int_{\varepsilon_{C}}^{\varepsilon_{A}}\sigma_{i}^{-}d\varepsilon_{i}\\ U_{hi}=U_{i}-U_{ei}=\int_{\varepsilon_{O}}^{\varepsilon_{A}}(\sigma_{i}^{+}-\sigma_{i}^{-})d\varepsilon_{i} \end{array}\right.$$

where *U_i_*, *U_ei_*, and *U_hi_* are the input energy, elastic energy, and dissipation energy during the *i*th cycle, respectively; *σ_i_*^+^ and *σ_i_*^-^ are the stress values on the loading path and unloading path, respectively; and *ε_A_*, *ε*_O_, and *ε_C_* are the strain values of point A, point O, and point C, respectively, which can be found in Figure 18.

*U_hi_* can be further divided into damage energy *U_hsi_* and damping energy *U_hzi_* because the rock is not continuous, isotropic, and homogeneous. On the one hand, *U_hsi_* is used to develop and extend the cracks, which causes plastic deformation; on the other hand, part of the energy is consumed by friction between crack surfaces and liquid viscosity, which can be deemed the damping feature of rock; thus, the energy is denoted *U_hzi_*. The area enclosed by the unloading curve of the *i*th cycle and the loading curve of the *i* + 1th cycle (the area of hysteretic loop *BCB* in Figure 18) can be regarded as *U_hzi_*, and the remaining area obtained by subtracting *U_hzi_* from *U_hi_* is *U_hsi_*. The expressions of *U_hsi_* and *U_hzi_* are presented in Equation (5).

(5) $$\left\{ \begin{array}{l} U_{hzi}=\int_{\varepsilon_{C}}^{\varepsilon_{B}}(\sigma_{i+1}^{+}-\sigma_{i}^{-})d\varepsilon_{i}\\ U_{hsi}=U_{hi}-U_{hzi}=\int_{\varepsilon_{O}}^{\varepsilon_{A}}(\sigma_{i}^{+}-\sigma_{i}^{-})d\varepsilon_{i}-\int_{\varepsilon_{C}}^{\varepsilon_{B}}(\sigma_{i+1}^{+}-\sigma_{i}^{-})d\varepsilon_{i} \end{array}\right.$$

#### 3.5.2. Assignment and Evolution Results of Energy

**Figure 19:**
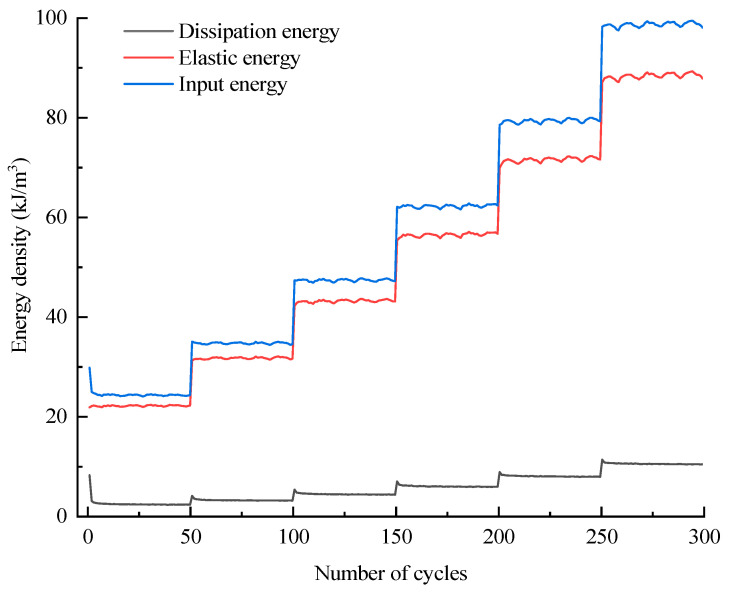
Assignment of input energy during the whole cyclic loading process.

**Figure 20:**
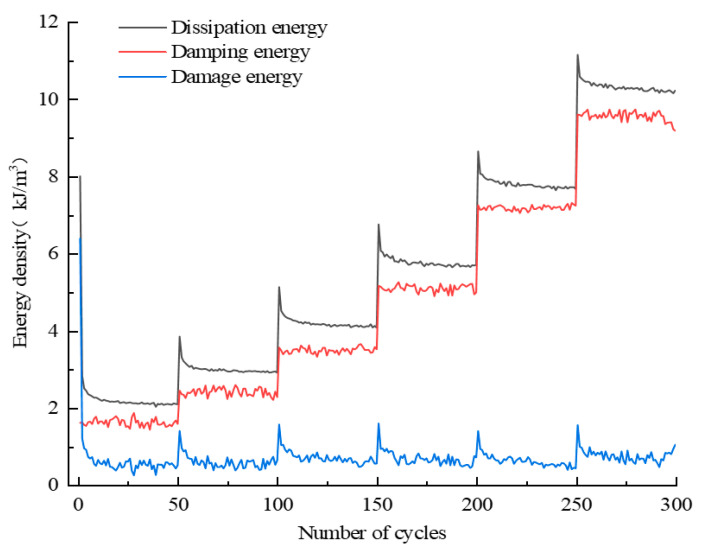
Assignment of dissipation energy during the whole cyclic loading process.

**Figure 21:**
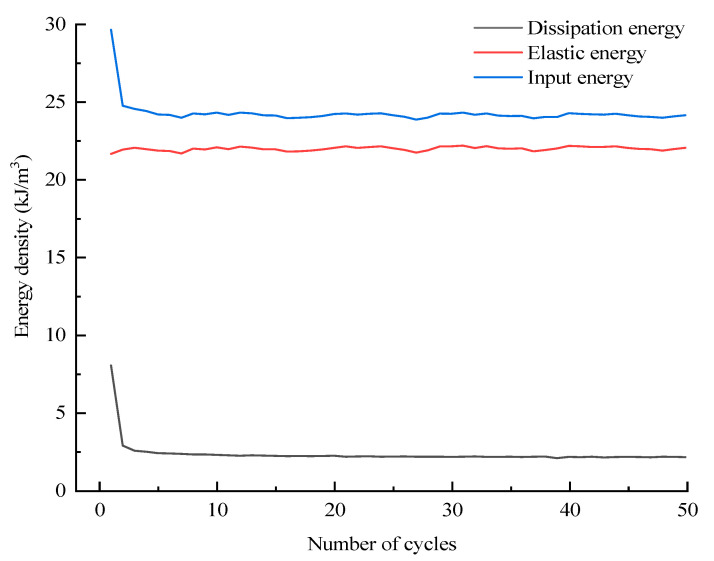
Assignment of input energy during the level I cycle.

**Figure 22:**
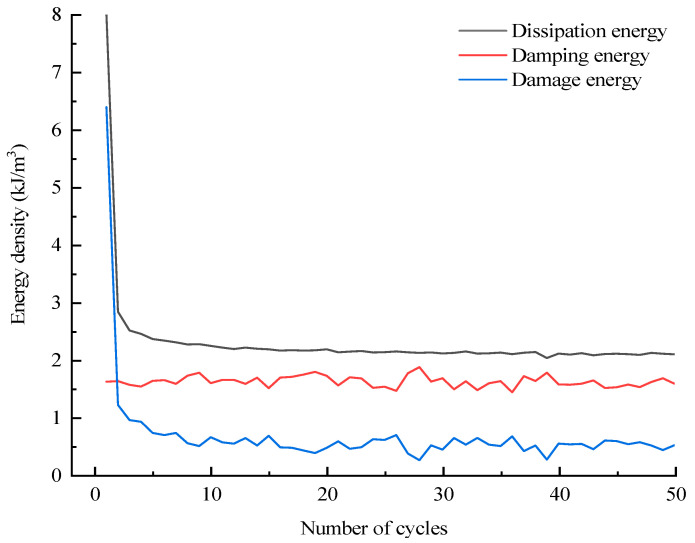
Assignment of dissipation energy during the level I cycle.

The assignment of total input energy during the whole cyclic loading process can be derived by Equation (4); likewise, the assignment of dissipation energy can also be derived by Equation (5). Taking the D10 specimen as an example, the corresponding results with regard to the assignments of *U_i_* and *U_hi_* are displayed in Figure 19 and Figure 20, respectively. In addition, the evolutions of various energies in level I are extracted from Figure 19 and Figure 20 to research the evolution law of energy for the case of constant-amplitude cyclic loading. The corresponding results are shown in Figure 21 and Figure 22.

The assignment of the total input energy is discussed first. Figure 19 and Figure 21 reveal that *U_i_* increases step by step with the stress level of the cycle and remains basically constant for equal amplitude cyclic loading of each stress level except for level I. The evolution curve of *U_i_* in level I exhibits an “L” shape, particularly because several initial cycles are devoted to compressing the initial cracks, which makes the specimen prone to irreversible deformation during the so-called compaction stage. The evolution characteristic of *U_ei_* is identical to that of *U_i_*; at each stress level, most of the energy of *U_i_* is stored by the specimen in the form of *U_ei_*, and the *U_ei_* of each cycle remains basically constant. *U_hi_* increases step by step with the stress level of the cycle, and the evolution of *U_hi_* in each stress level also presents the “L” shape because numerous new cracks are generated during the first loading of each stress level, which will consume additional energy compared to the subsequent cycles.

The assignment of *U_hi_* is discussed next. Figure 20 and Figure 22 reveal that *U_hzi_* presents a similar evolution law to *U_ei_*, i.e., *U_hzi_* has a step-like increasing trend in general and remains relatively stable at each stress level. This is attributed to the fact that *U_hzi_* is closely related to the existence of cracks, and the increasing stress level will cause large new cracks to develop; thus, the *U_hzi_* of the latter stress level is significantly larger than that of the previous level. As the equal amplitude cyclic loading of each stress level causes only slight cracks, the *U_hzi_* at each stress level is stable. In terms of *U_hsi_*, the value is small in most cycles in addition to the first cycle of each stress level because the development of new cracks in the first cycle of each stress level requires a large *U_hsi_*.

#### 3.5.3. Damage Evolution Based on Energy Analysis

**Figure 23:**
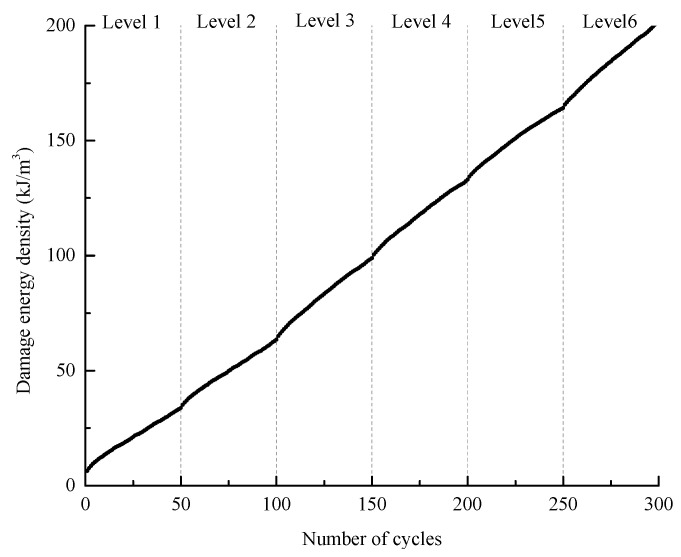
Evolution of the total damage energy during the whole cyclic loading process.

**Figure 24:**
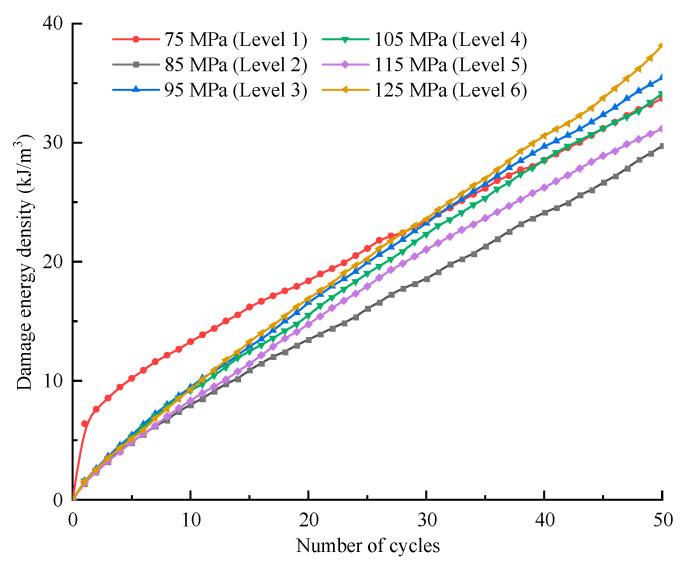
Evolution of the total damage energy during each stress level (*σ*_3_ = 10 MPa).

According to the definition of each type of energy, *U_hsi_* can reflect the damage degree of rock in each cycle; thus, the damage variation during the entire deformation process can be indicated by the accumulation of *U_hsi_* (Figure 23), and the damage evolution of the specimen within each stress level is plotted in Figure 24.

Figure 22 and Figure 23 show that the damage increases continuously in general, while at each stress level, the increase exhibits a law of deceleration-stabilization, especially at level I. Regarding the last several cycles of level VI, the damage exhibits a trend of acceleration, which indicates that the specimen is approaching failure. The whole damage variation is in good agreement with the process of irreversible strain increase, i.e., the initial cracks are compressed first, and then secondary cracks are generated and extended. When the stress approaches the compressive strength of the specimen, the cracks develop quickly and coalesce.

### 3.6. Failure Mode of Phyllite

**Figure 25:**
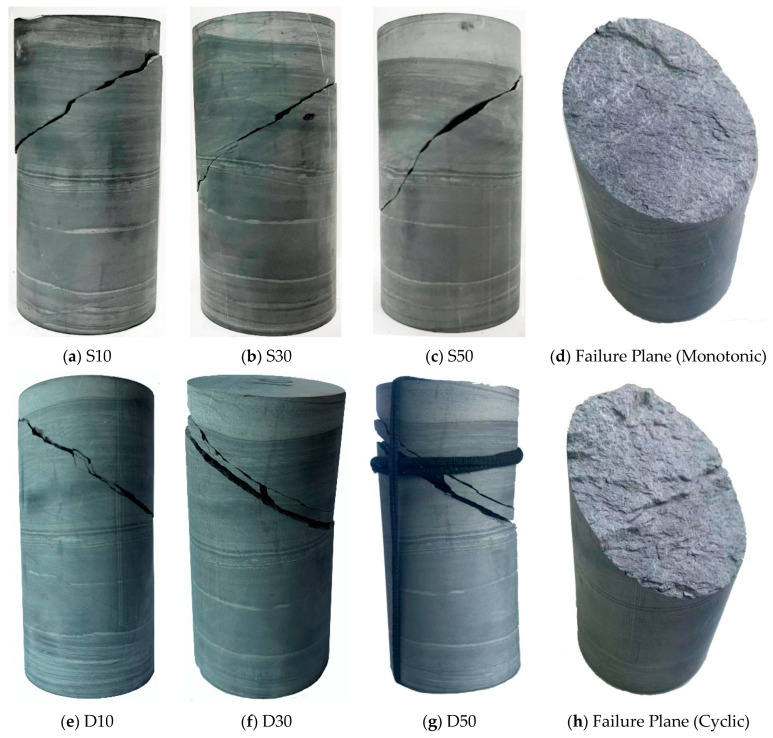
The failure modes of typical specimens.

**Figure 26:**
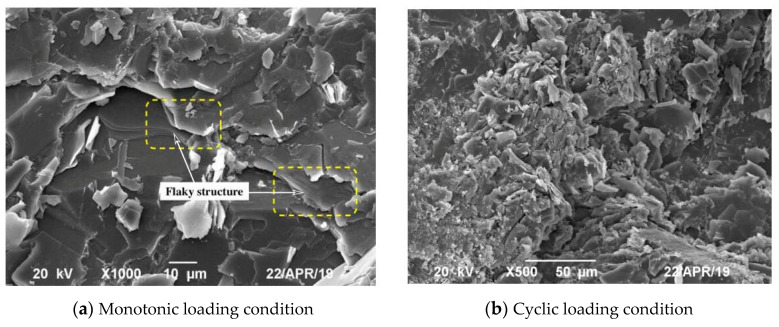
SEM images of phyllite after rock failure.

The failure mode of rock can reflect the failure development process of rock to a certain extent. Here, the failure mode is analysed by combining the macroscale and microscale perspectives, the macroscopic fracture surfaces of the specimens subjected to monotonic loading and cyclic loading are shown in Figure 25, and the microstructures of fracture surfaces obtained by SEM method are shown in Figure 26. Here, specimen D50 is chosen for the micromechanical analysis because the specimen faced with the most severe loading condition; as a result, the micro characteristics are relatively obvious.

Figure 25 reveals that broken specimen presents an inclined-shear failure mode whether under monotonic or cyclic loading. The macroscopic shear crack starts from a weak surface in the middle of the specimen and extends obliquely, but it does not penetrate the end surface of the specimen. This is due to the small influence of the end-restraint effect and the large influence of Poisson’s effect. In contrast to monotonic loading, the specimen under cyclic loading presents greater fragmentation degree, which is indicated by the large amount of debris and powdery particles along the macroscopic shear plane. This is because the cyclic loading causes repeated friction on the internal structure surface, which destroys the original internal structure, the bonding force between particles is weakened, resulting in more holes, cracks, and failure surfaces. Therefore, the discrete rock particle is the peculiar characteristic in terms of the cyclic loading condition; the failure mode can be considered combination of shear failure and fatigue failure.

The microscopic characteristics of shear surface also reveal the difference between the failure modes under monotonic and cyclic loading. For the case of monotonic loading (Figure 26a), the shear surface exhibits a flaky structure, so the intergranular failure occurs at the micro level. The multiple scaly layers comprise the macroscopic fracture surface, which implies shear failure. For the case of cyclic loading (Figure 26b), the crushed phyllite grains were observed between the fracture surfaces, so the transgranular failure occurs at the micro level. The macroscopic fracture surface accompanied by crushed grains imply shear failure and fatigue failure.

From the perspective of the microscopic and macroscopic failure morphology, it can be seen that the crack growth process caused by cyclic loading is irreversible. The failure mechanisms of monotonic and cyclic loadings are inconsistent, and failure of the specimen caused by cyclic loading is more serious. In the entire damage development process of the phyllite under cyclic loading, the specimen shows the continuous accumulation of irreversible strain on the macro scale. The irreversible strain arises from the shear behaviour and grain-crushing behaviour, the former mainly occurs in the first few cycles of each stress level, while the latter mainly occurs in the subsequent constant-amplitude cycles of each stress level.

## 4. Discussion

In recent years, the construction demand for high-speed railways in China has increased, and high-speed railway tunnel construction is a key part of these projects. The load form of a high-speed railway is complex, and it may pass through phyllite strata. Carbonaceous phyllite is weak, easily softens in water and has poor mechanical properties. Tunnels constructed in carbonaceous phyllite surrounding rock are prone to suffer from issues, such as the uplifting of the inverted arch, lining cracks, and even collapse under cyclic loading, which had a serious impact on the safety of high-speed railways. Therefore, a multistage constant-amplitude cyclic loading experimental scheme is designed to analyse the deformation characteristics and damage evolution of carbonaceous phyllite under triaxial cyclic loading.

**Figure 27:**
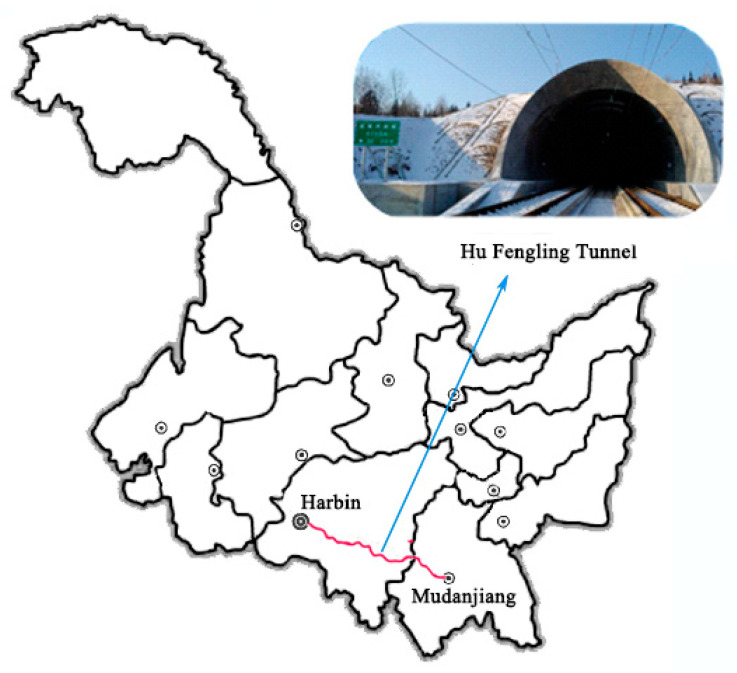
Location of the Hufengling Tunnel in Heilongjiang Province.

Although the number of cyclic loading tests on phyllite specimens is limited, the results can be a reference for tunnel engineering applications. Take the Hufengling Tunnel mentioned above as an example. The location of the Hufengling Tunnel, which is an 8755 m long high-speed railway tunnel with a design speed of 250 km/h (Figure 27). The lithology of some strata where the tunnel crosses is phyllite, and the rock mass is developed with multidirectional bedding. Because of the high-speed and multifrequency trains, the tunnel surrounding rock will be damaged and destroyed under long-term cyclic loading. According to the experimental results achieved in this paper, some measures can be taken to relieve the rock damage and reduce possible surrounding rock issues.

**Figure 28:**
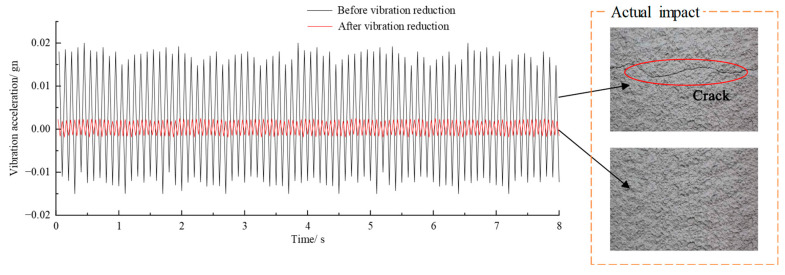
Variation curve of the measured vibration acceleration with time of the tunnel base.

Referring to the results above, the *E_S_* and stiffness of phyllite will decrease when the applied stress is greater than the critical value. Therefore, it is suggested that vibration reduction measures should be taken when designing the bottom structure of a high-speed railway tunnel to reduce the peak value of train loading that is transmitted to the surrounding rock of the tunnel base to a reasonable range, such as the commonly used elastic foundation, open trench, filled trench [40], and wave impeding barrier (WIB) methods [41]. According to the experimental results in this paper, it is appropriate to guarantee that the loading amplitude of the surrounding rock of the tunnel base is approximately 50% of the monotonic compressive strength. A test section in the Hufengling Tunnel is selected for vibration reduction research and obtained the variation curves of the measured vibration acceleration with time in terms of the surrounding rock of the tunnel base before and after the vibration reduction. The results are shown in Figure 28. The vibration acceleration decreases significantly after vibration reduction, which is conducive to reducing the loading that is transmitted to the surrounding rock.

**Figure 29:**
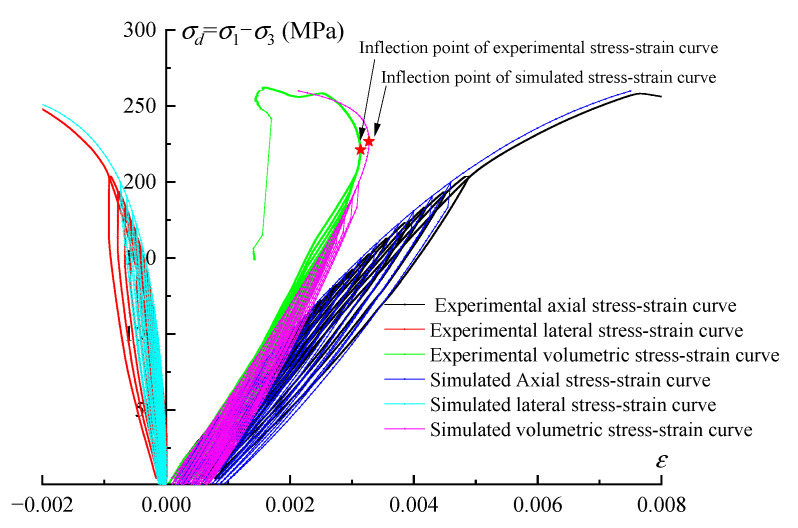
The multistage constant-amplitude cyclic loading constitutive model of rock under cyclic loading proposed by Hu et al. [42].

In terms of tunnel stability and safety analysis, the tunnel is faced with cyclic loading of the train for a long time, and irreversible deformation accumulation and energy loss of the surrounding rock is inevitable; that is, damage to the surrounding rock is inevitable. Therefore, the corresponding damage analysis is needed, which relies on the constitutive model in numerical simulation. The deformation behaviour recognition and damage evolution analysis conducted in this paper are beneficial to the constitutive model establishment, including the variation law and parameters determination; the detailed simulation method can be found in Hu et al. [42], and the relevant simulated results are shown in Figure 29, in which the high degree of coincidence between simulated irreversible strain and experimental irreversible strain proves the correctness of damage evolution explored in this paper. On this basis, the corresponding secondary development can be developed to analyse the changes in the stress and deformation of the surrounding rock; in this way, the remaining life of the structure can be estimated. The corresponding model is a direction of further research.

In addition, it should be noted that the test was carried out on intact carbonaceous phyllite in this paper. However, natural rock is always heterogeneous, containing joints, cracks, and beddings located in shear zones with certain levels of damage. For example, Zhang et al. [43] and Fu et al. [44] studied the influence of the strength change of slate under different bedding angles, and carbonaceous phyllite is similar to slate, which has a bedding structure. Therefore, it is necessary to consider the different bedding angles of the specimens for cyclic loading tests to more reasonably assess the stability and safety of high-speed railway tunnels, which will be improved in the next work.

## 5. Conclusions

This paper carried out triaxial multistage constant-amplitude cyclic loading test on a carbonaceous phyllite sample. The relevant findings are applicable to similar soft rocks, and the specific conclusions are summarized below.

(1) *E_S_* and *υ* of rock vary with the stress level and the constant-amplitude cycles. With the increase in stress level, the *E_S_* will decrease in a step-like form, while *υ* will increase in a step-like form. In addition, as constant-amplitude cycles develop, the *E_S_* will decrease approximately linearly, while *υ* will increase slowly, and the rise of the stress level will accelerate their change rates.
(2) The *ε^irr^* generated in each stress level consists of two parts. One part is the rapid accumulation in the first few cycles, which is caused by the rise in stress levels. Another part is the gradual accumulation in the remaining cycles, which is caused by multiple constant-amplitude cycles. The relationship between stress level and *ε*_1_*^irr^* can described by an exponential function.
(3) The $\varepsilon_{v}^{iir}$ changes nonmonotonically. At a lower stress level, the increment of $\varepsilon_{v}^{iir}$ is positive, and rock specimen is in a compaction stage. With the increase of stress level, the increment of $\varepsilon_{v}^{iir}$ will become negative, and the stage of the specimen will change from compaction to dilation.
(4) The rock damage can be reflected by cumulative damage energy. With the development of constant-amplitude cycles at most stress levels, the growth rate of damage changes from fast to slow and finally stabilizes. However, as the stress level approaches the compressive strength, the damage accumulation exhibits a pattern of deceleration, stabilization, and then acceleration.
(5) The damage of a specimen under monotonic loading is dominated by shear behaviour, which implies that the corresponding failure mode is shear failure. By contrast, the specimen under cyclic loading presents macroscopic shear surfaces and crushed grains, which implies that the corresponding failure mode is the combination of shear failure and fatigue failure.

## Figures and Tables

**Table 1:** Physical and mechanical parameters of carbonaceous phyllite specimen.

*ρ* (g/cm^3^)	*E_S_* (Gpa)	*υ*	*c* (kPa)	*φ* (°)	*σ_c_* (MPa)
2.33	13	0.14	103.97	31.67	111.32

**Table 2:** Mechanical parameters of the specimens under monotonic loading.

Specimen No.	*σ*_3_ (MPa)	*σ_f_* (MPa)	*ε_f_* (10^−3^)	*E* (GPa)	*υ*
S10	10	166.67	6.14	28	0.04
S30	30	208.20	6.95	39	0.15
S50	50	227.38	6.26	47	0.21

**Table 3:** Different parameters of the triaxial cyclic compression.

Specimen	*σ*_3_ (MPa)	*σ_min_* (MPa)	*σ_max_* (MPa)
D10	10	25	75, 85, 95, 105, 115, 125
D30	30	25	145, 155, 165, 175, 185, 195
D50	50	25	162, 172, 182, 192, 202, 212

## Data Availability

All data are available from the authors.
